# Hagfish olfactory repertoire illuminates lineage-specific diversification of olfaction in basal vertebrates

**DOI:** 10.1016/j.isci.2025.114118

**Published:** 2025-11-19

**Authors:** Hirofumi Kariyayama, Yusuke Ooi, Hiromu Kashima, Taiki Nakanowatari, Riho Harada, Yoko Yamaguchi, Daichi G. Suzuki

**Affiliations:** 1 Graduate School of Comprehensive Human Sciences, University of Tsukuba, Tsukuba, Japan; 2 Laboratory for AI Biology, RIKEN Center for Biosystems Dynamics Research, Kobe, Japan; 3 Graduate School of Life and Environmental Sciences, University of Tsukuba, Tsukuba, Japan; 4 Graduate School of Natural Science and Technology, Shimane University, Shimane, Japan; 5 School of Life and Environmental Sciences, University of Tsukuba, Tsukuba, Japan; 6 Institute of Agricultural and Life Sciences, Academic Assembly, Shimane University, Shimane, Japan; 7 Institute of Life and Environmental Sciences, University of Tsukuba, Tsukuba, Japan

**Keywords:** Evolutionary biology, Genomics, Molecular biology, Zoology

## Abstract

Chemosensory receptors for olfaction of vertebrates have undergone remarkable diversification, but the ancestral repertoire remains incompletely understood. Here, we present a comprehensive genome-wide survey and gene expression analyses of the olfactory repertoire in hagfish, a cyclostome group that typically inhabits the deep sea with a well-developed olfactory system. We identified 48 olfactory receptors (ORs), two vomeronasal type 1 receptors (V1Rs), 135 typical vomeronasal type 2 receptors (V2Rs), and no trace amine-associated receptors (TAARs) in the inshore hagfish, *Eptatretus burgeri*. Expression analysis confirmed that most of these genes are expressed predominantly in the olfactory organ, supporting that these receptors are functional. Our findings suggest that typical olfactory V2Rs were present in the common ancestor of all vertebrates. This study highlights the evolution of vertebrate olfaction in a lineage-specific manner, underscoring the importance of hagfish as a key animal for reconstructing the evolutionary history of early vertebrates.

## Highlights

• Genomic and expression analyses reveal hagfish olfactory repertoire
• V2Rs are extensively diversified in hagfish
• Olfactory function of V2Rs is traced back to the common ancestor of vertebrates
• Olfactory repertoires of basal vertebrates underwent lineage-specific evolution

## Graphical abstract

**Figure:**
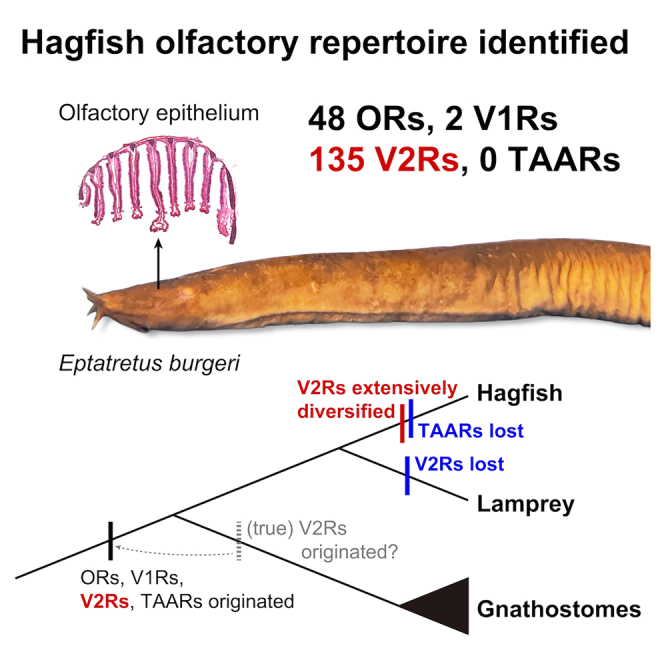

## Introduction

Olfaction is a vital sense modality for animals. In particular, it plays an even more important role in darkness because visual information is limited in such environments. It is suggested that our mammalian ancestors experienced nocturnal life, in which they heavily relied on olfaction, accompanied by enlargement of the olfactory bulb and cerebral cortex.^1^ Along with this brain evolution, their olfactory repertoire genes diversified explosively.^2^

A similar evolutionary trend may have occurred to hagfish, which belongs to the basal-most lineage of vertebrates (i.e., the cyclostomes). Hagfish typically live in light-decaying deep water and have a degenerate visual system.^3^ Instead, these animals possess a highly developed olfactory bulb and telencephalon (the brain region homologous to the mammalian cerebrum), suggesting that their capacity for olfaction is correspondingly elaborated.^4^ However, the repertoire of hagfish chemosensory receptors for olfaction remains largely unknown.

Apart from such habitat-specific diversification of olfaction, hagfish may provide crucial information for the early evolution of the vertebrate olfactory repertoire. Within vertebrates, there are four major gene families: olfactory receptors (*OR*s), type 1 vomeronasal receptors (*V1R*s), type 2 vomeronasal receptors (*V2R*s), and trace amine-associated receptors (*TAAR*s), all of which encode G-protein coupled receptors (GPCRs).^5^ Furthermore, *OR*s are divided into two major subfamilies: type 1 and type 2.^6^ Type 1 *OR*s are dramatically expanded in the tetrapod lineage, while some type 2 *OR*s are suggested to be non-olfactory.^6^ In general, ORs are known to detect volatile odorants and amino acids; V1Rs respond to small, relatively volatile molecules including steroid hormones; V2Rs are activated by amino acids and polypeptides; and TAARs respond to trace amines.^5^^,^^7^ Previous research has shown that the lamprey genome contains 27 *OR*s (the sea lamprey, *Petromyzon marinus*),^8^ six *V1R*s (the sea lamprey and the Arctic lamprey, *Lethenteron camtschaticum*),^9^ one (the sea lamprey) or two (the Arctic lamprey) *V2R* candidates, and 32 (the sea lamprey) or 51 (the Arctic lamprey) *TAAR*s.^10^ However, Kowatschew and Korsching^9^ report that the sea lamprey *V2R* candidate gene is not expressed in the olfactory epithelium and does not appear to be a true, functional V2R; therefore, the authors refer to it as “V2R-like.” Bi et al.^11^ identified more than 50 *V2R* candidate sequences in hagfish. Still, Zhang et al.^12^ suggest that no typical *V2R*s exist in extant agnathans (i.e., lampreys and hagfish) because these cyclostome *V2R* candidates did not form a clade with known *V2R*s in their phylogenetic analysis. To understand the ancestral olfactory repertoire of vertebrates, it is therefore necessary to investigate the hagfish olfactory repertoire in a more thorough and detailed manner.

In this study, we conducted a comprehensive genome-wide survey and gene expression analyses of hagfish chemosensory receptors for olfaction. We first identified 48 *OR*, two *V1R*, 137 *V2R*, but no *TAAR* candidate sequences in the inshore hagfish (*Eptatretus burgeri*) genome. Then, phylogenetic analysis revealed that all *OR* candidates are type 1 *OR* genes and that the two *V1R* candidates are also true vertebrate *V1R*s. Regarding *V2R*s, 135 among 137candidates were estimated to be typical *V2R*s. We next explored the genomic distribution of the identified olfactory repertoire genes and found that phylogenetically close genes tend to be clustered in the genome. Transcriptome analysis indicated that most of these olfactory repertoire genes are expressed in the olfactory organ. Using *in situ* hybridization, we further confirmed that at least some of these genes are expressed in olfactory sensory neurons. These results reveal the comprehensive olfactory repertoire of hagfish, suggesting that the common ancestor of vertebrates had functional olfactory V2Rs and that both lampreys and hagfish have experienced lineage-specific diversification of their olfactory repertoires.

## Results

### Gene identification and phylogenetic analysis of the hagfish olfactory repertoire

#### ORs

**Figure 1:**
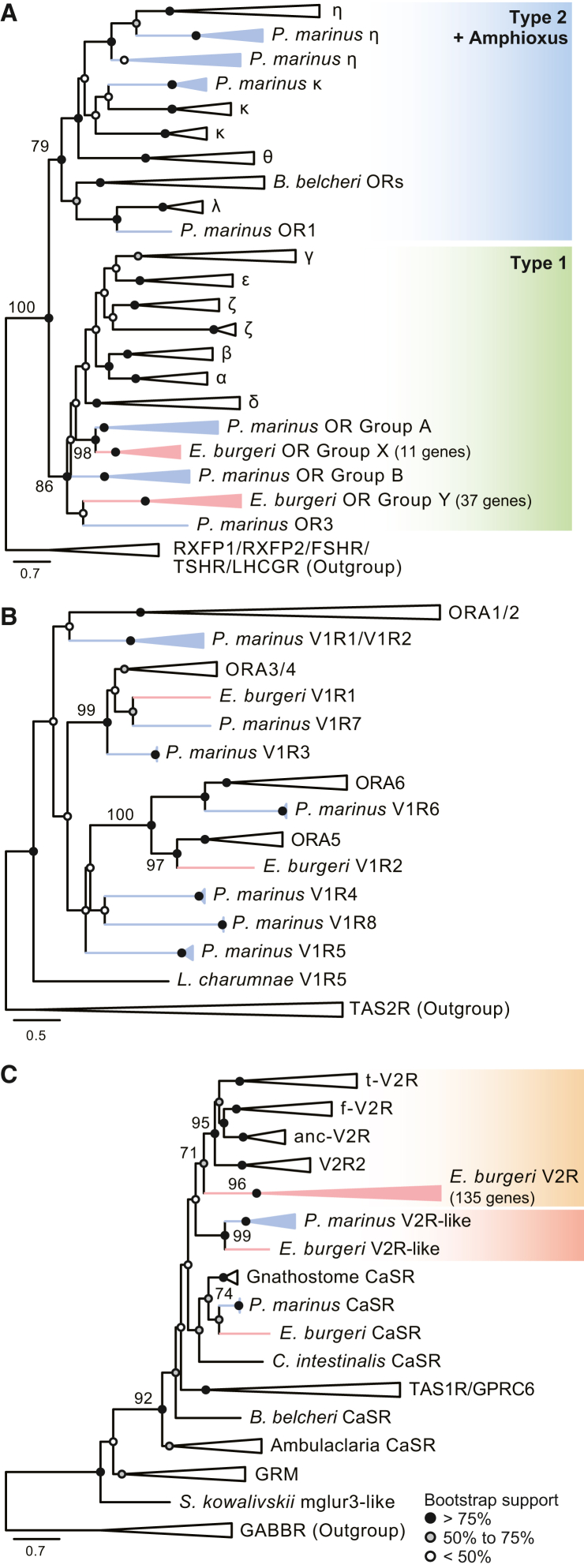
Phylogenetic analysis of OR, V1R, and V2R amino acid sequences (A) Phylogenetic tree of ORs. White triangles indicate clades of type 1 OR subgroups of gnathostomes, type 2 OR subgroups of gnathostomes, amphioxus ORs, and outgroup genes. (B) Phylogenetic tree of V1Rs. White triangles indicate subgroups of gnathostome V1R genes and outgroup genes. (C) Phylogenetic tree of V2Rs. White triangles indicate subgroups of gnathostome V2Rs, gnathostome CaSRs, gnathostome TAS1R/GPRCs, Ambulacraria CaSRs, GRMs, and outgroup sequences. For all trees, red triangles and edges denote clades of inshore hagfish (*E. burgeri*) genes, while blue triangles and edges mark clades of lamprey (*P. marinus* and/or *L. camtschaticum*) genes. The range of bootstrap support values is shown as the circle at each node (black >75, 75 ≥ gray ≥50, white <50). The numbers described on some nodes indicate exact bootstrap values. Scale bar represents the number of amino acid substitutions per site. For the list of genes used, see Table S3. For more detailed trees, see Data S1, S2, and S3.

In osteichthyes, including tetrapods, *OR*s are the biggest gene family among olfactory repertoire genes (see Table 1). We explored hagfish gene models and genome data and then found 48 *OR* candidates (for the list of the identified inshore hagfish olfactory repertoire genes, including these *OR* candidates, see Table S1). We also identified 62 sea lamprey *OR* genes that are predicted to be functional from the newest version^13^ of the sea lamprey genome (for the list of the sea lamprey olfactory repertoire genes identified in this study, see Table S2). Phylogenetic analysis revealed that all of these inshore hagfish genes form two groups, both belonging to the type 1 OR subfamily (Figure 1A). Each group contained inshore hagfish genes alone, showing monophyly with a high support value (100%). One of them (inshore hagfish Group X) included 11 genes, belonging to a larger group with a monophyletic sea lamprey clade (sea lamprey Group A) supported by a high bootstrap value. The other (inshore hagfish Group Y) consisted of 37 genes and formed a monophyletic group with a sea lamprey gene, with a relatively low support value. No type 2 *OR* genes were identified from inshore hagfish, although some sea lamprey genes were included in this subfamily as previous research suggested.^6^

**Table 1:** Comparison of olfactory repertoires in vertebrates

	OR	V1R	V2R(-like)	TAAR
Inshore hagfish (*Eptatretus burgeri*)	48^a^	2^a^	135^a^ (1^a^)	0^a^
Sea lamprey (*Petromyzon marinus*)	62^a^/27^8^	8^a^/4^8^/6^9^	0^8^ (1^a^^,^^9^)	28^8^/32^10^
Elephant shark (*Callorhinchus milii*)	8^14^	6^14^	30^12^	5^10^
Zebrafish (*Danio rerio*)	154^6^	7^15^	72^12^	18^10^
Western clawed frog (*Xenopus tropicalis*)	824^6^	15^15^	691^12^	5^16^
Mouse (*Mus musculus*)	1,035^6^	239^17^	154^12^	9^16^

a This study.

#### V1Rs

*V1R* or olfactory receptor class A (*ORA*) genes are known to be diversified in sarcopterygians, including tetrapods, in which these receptors are thought to be involved in pheromone detection.^18^ We found two *V1R* candidates in the inshore hagfish genome, while more *V1R*s (eight genes) were identified in the sea lamprey genome. It is suggested that vertebrate *V1R*s are divided into six subfamilies (*ORA1*–*6*).^15^ Through phylogenetic analysis, we found that the inshore hagfish V1R1 candidate forms a monophyletic clade with gnathostome ORA3/4 along with a sea lamprey V1R, supported by a high bootstrap value (99%; Figure 1B). The inshore hagfish V1R2 candidate was placed as the sister group of the ORA5 family of gnathostomes with a high bootstrap value (97%). In addition, some sea lamprey V1Rs constructed a clade with gnathostome ORA1/2 with a relatively high support value, although the phylogenetic positions of other sea lamprey genes were unclear due to their relatively low support values.

#### V2Rs

V2Rs are chemoreceptors targeting amino acids and peptide pheromones in gnathostomes.^19^^,^^20^^,^^21^^,^^22^^,^^23^ We identified 137 *V2R* candidates in the inshore hagfish. In contrast, only one *V2R-like* gene was found in the sea lamprey genome, consistent with previous reports.^9^ Phylogenetic analysis revealed that one inshore hagfish V2R candidate forms a clade with lamprey and gnathostome calcium-sensing receptors (CaSR), another grouped with the lamprey V2R-like sequences, and the remaining 135 V2R candidates form a monophyletic clade together with high bootstrap value (96%), being situated in a basal position to gnathostome V2Rs more closely than the cyclostome V2R-like clade (Figure 1C). Based on these results, we estimated the first and second candidate genes as the inshore hagfish *CaSR* and *V2R-like*, respectively, and the last group containing 135 candidates as typical *V2R*s.

#### TAARs

Previous studies have reported that *TAAR*s are diversified in lamprey.^8^^,^^10^ To find hagfish *TAAR*s, we first performed a reciprocal BLAST search against inshore hagfish gene models, but no plausible candidates were found. For further confirmation, we performed phylogenetic analysis of top hit inshore hagfish sequences and known *TAAR*s. Consequently, these inshore hagfish genes did not show affinity to TAARs but to various groups of GPCRs, including the 5-hydroxytryptamine receptor 4 (5-HT4 or HTR4), adrenoceptor beta 1, and dopamine receptor family (Data S4). These results suggest that the inshore hagfish have no *TAAR* homologs, consistent with previous research.^24^

### Genomic distribution of the hagfish olfactory repertoire genes

To clarify the diversification pattern of the hagfish olfactory repertoire, we then examined the genomic distribution of the identified genes using the following analyses.

First, we defined phylogenetically related clades for ORs and V2Rs (see STAR Methods). We categorized three major clades for ORs; Group X corresponded entirely to Clade 1, while Group Y was subdivided into Clade 2 and 3 (see Figure S1). For V2Rs, nine major clades (Clade 1–9) were distinguished, and the remaining unclassified genes were categorized into “Else” (see Figure S2).

**Figure 2:**
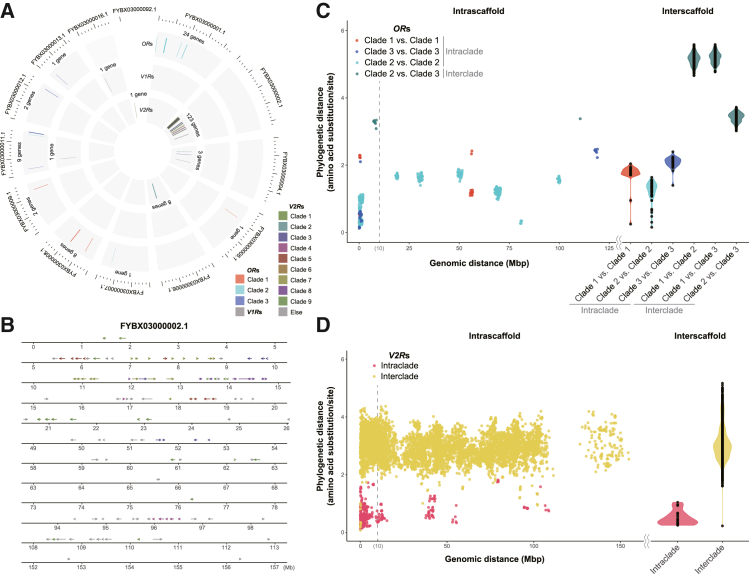
Genomic distribution of the hagfish olfactory repertoire genes (A and B) Genomic mapping of the olfactory repertoire genes to the scaffolds larger than 0.1 Mbp (A) and to a single scaffold, FYBX03000002.1 (B). Each clade of *OR* and *V2R* genes (for identification of clades, see text) is differently colored. Scale interval: 10 Mbp for (A). (C and D) Plots (left, scatterplots for intrascaffold gene pairs; right, violin plots for interscaffold gene pairs) between phylogenetic and genomic distances, which are calculated between each pairwise combination of the identified genes: *OR*s (C) and *V2R*s (D).

Next, we mapped all identified olfactory repertoire genes onto the genome (Figure 2A). As the current inshore hagfish genome is not assembled at the telomere-to-telomere level, we arranged scaffolds (>0.1 Mbp) from longer to shorter. As an overall pattern, the olfactory repertoire genes exhibited a patchy distribution. For example, *OR*s in the same clade tended to co-occur on the same scaffold. Even in each scaffold, phylogenetically related genes tended to be neighboring closely in the genome as follows. We calculated phylogenetic and genomic distances between each pairwise combination of the identified genes and then made scatter (for intrascaffold gene pairs) and violin (for interscaffold gene pairs) plots between these two distances (Figure 2C). As a result, we found that a higher portion of intraclade pairs (32.2%; 140/435 pairs) were located within a shorter genomic distance (<10 Mbp), compared to the interclade pairs (1.0%; 7/693 pairs).

*V2R*s also exhibited a patchy genomic distribution, and most of them (91.1%; 123/135) were concentrated on a single scaffold (FYBX03000002.1). In this scaffold, phylogenetically close genes were arranged in clusters along the genome (Figure 2B). In addition, scatter and violin plotting between phylogenetic and genomic distances (Figure 2D) showed that most of the intraclade pairs (69.8%; 273/391 pairs) were located within a shorter genomic distance (<10 Mbp), but fewer interclade pairs (21.5%; 1862/8652 pairs) were within such a close distance.

In teleosts, *V2R* genes are known to be clustered in one particular chromosomal region between two landmark genes, phospholipase C eta (*Plch1*) and membrane metallo-endopeptidase mme (*Mme*)/neprilysin (*Nep*).^12^^,^^25^ We thus performed microsynteny analysis and found that these landmark genes are not located in FYBX03000002.1 but in other scaffolds that contain no or substantially distant (>50 Mbp) *V2R* genes (Figure S3; Table S4). A similar microsynteny was observed in the sea lamprey, suggesting that this pattern is specific in the cyclostome lineage.

### Organ-specific gene expression of the hagfish olfactory repertoire genes

For the examination of whether the hagfish olfactory repertoire gene candidates are indeed expressed in the olfactory organ, we performed organ-specific gene expression analyses using bulk RNA sequencing (RNA-seq) data (the olfactory organ, brain, pituitary gland, gill, intestine, kidney, liver, testis, and ovary). As a result, we confirmed that most of these candidate genes were highly expressed in the olfactory organ.

**Figure 3:**
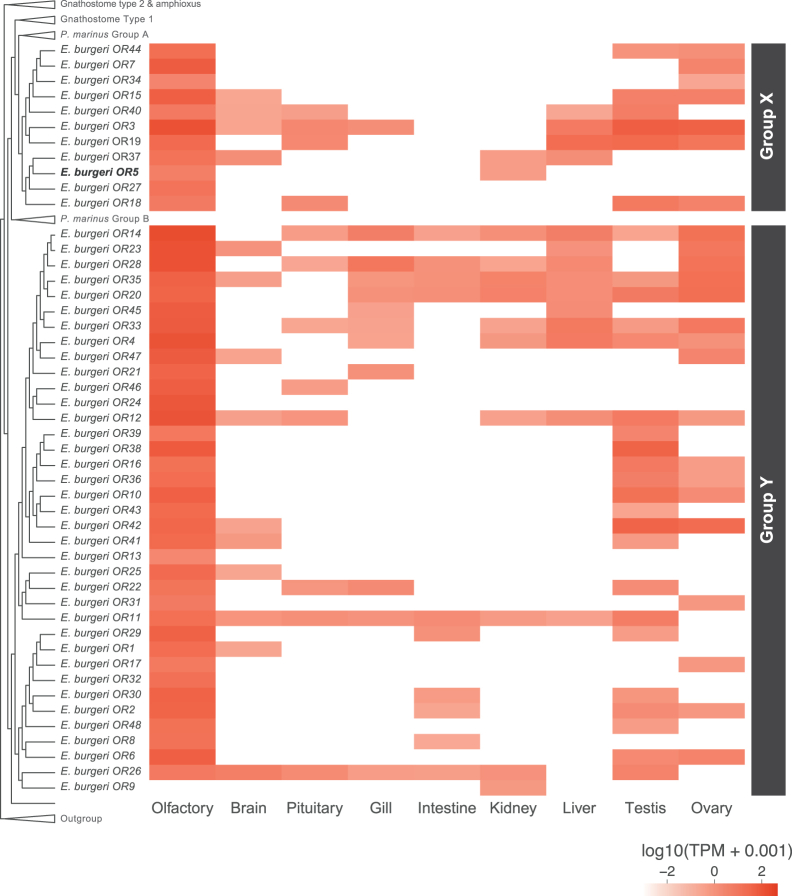
Gene expression profile of *OR* candidates across various organs Expression levels are shown as TPM values, log-transformed. Genes on the *y* axis are ordered according to the phylogenetic tree shown in Figure 1. The *OR* gene used for *in situ* hybridization analysis (*E. burgeri OR5*) is boldfaced.

First, 47 genes among 48 *OR* candidates showed high expression levels in the olfactory organ (44 genes showed the highest expression levels in the olfactory organ among the nine tested organs), suggesting their function as true olfactory receptors (Figure 3). Notably, many (19 genes) are also found to be expressed at a certain level in the testis and/or ovary.

**Figure 4:**
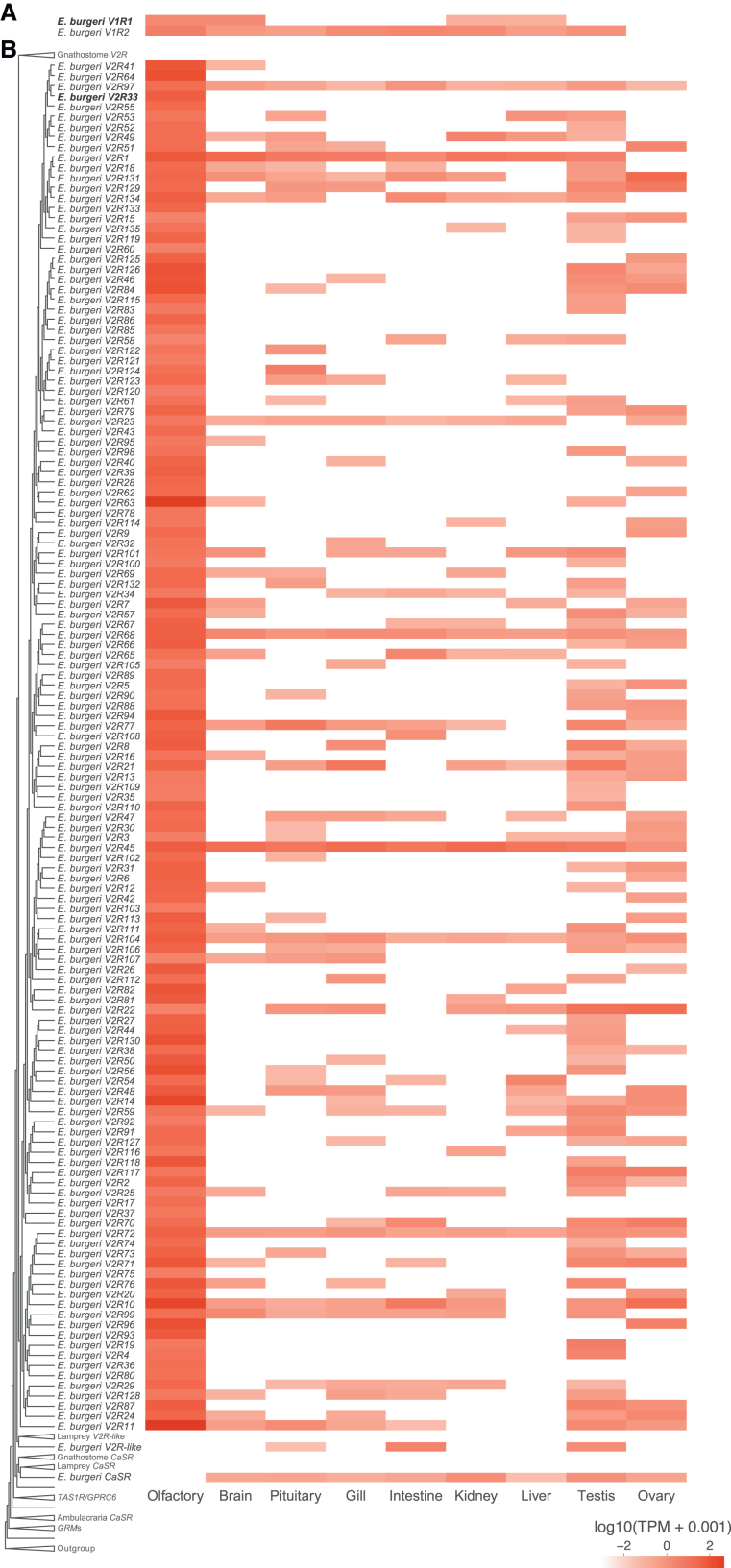
Gene expression profile of organ-specific differential expression analysis for *V1R* and *V2R* candidates across various organs (A) *V1R* candidates, (B) *V2R* candidates. Expression levels are shown as TPM values, log-transformed. Genes on the *y* axis are ordered according to the phylogenetic tree shown in Figure 1. The genes used for *in situ* hybridization analysis (*E. burgeri V1R1* and *V2R33*) are boldfaced.

Second, we confirmed that the two *V1R* candidates were also expressed at a relatively high level (transpripts per million, TPM >1) in the olfactory organ (Figure 4A). The inshore hagfish *V1R1* gene was also expressed in the brain at the same expression level and was slightly weakly expressed in the liver and kidney. The inshore hagfish *V1R2* gene showed the highest expression in the olfactory organ, with lower expression levels in other organs.

Last, we detected expression of all 135 inshore hagfish putative *V2R* genes in the olfactory organ (Figure 4B). Moreover, almost all of them (134 genes except *V2R22*) showed the highest expression in this organ. Impressively, expression of these genes was also frequently detected in the testis and/or ovary. In contrast, neither the inshore hagfish *CaSR* nor *V2R-like* was expressed in the olfactory organ; instead, the former was expressed in all the other organs tested (especially in the kidney and testis), while expression of the latter was detected highly in the intestine and testis, and weakly in the pituitary gland.

### Gene expression patterns of the hagfish olfactory repertoire genes in the olfactory epithelium

To investigate whether the olfactory repertoire gene candidates are specifically expressed in olfactory sensory neurons, we performed *in situ* hybridization analysis for an inshore hagfish OR (*OR5*), a V1R (*V1R1*), and a V2R (*V2R33*) gene, selected based on their expression levels and predicted sequence lengths.

**Figure 5:**
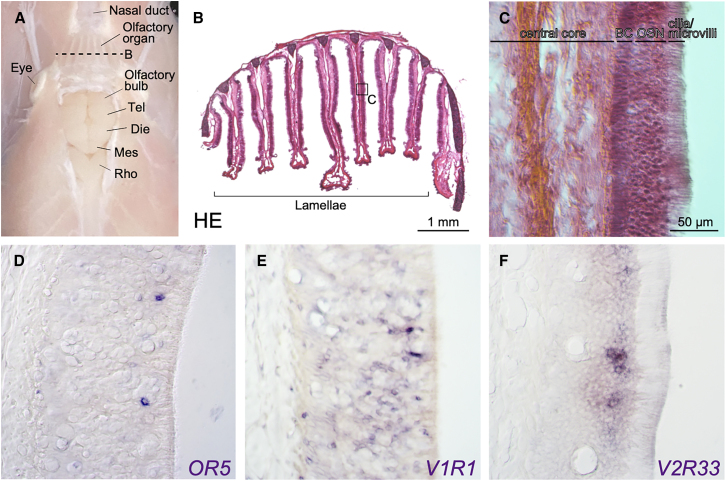
Histological analysis of the hagfish olfactory organ and gene expression patterns of olfactory repertoire gene candidates (A) Overview of the hagfish olfactory organ and brain, dorsal view. The skin and dorsal roof of the braincase are removed. The section plane for (B) is indicated by the dashed line. (B) Cross-section of the olfactory organ, HE staining. (C) Magnified image of (B), showing the layered structure of the olfactory lamina. (D–F) Gene expression patterns of *E. burgeri OR5* (D), *V1R1* (E), and *V2R33* (F). BC, basal cells; Die, diencephalon; Mes, mesencephalon; ORC, olfactory sensory neuron; Rho, rhombencephalon; Tel, telencephalon. The scale bar for (C–F) is shown in (C).

The hagfish olfactory organ, or the nasal basket, is situated anterior to the brain (Figure 5A).^26^^,^^27^ It consists of a series of seven olfactory lamellae, oriented parallel to the body axis and attached to the dorsal roof of the olfactory cavity (Figure 5B).^28^

By histological analysis, we first confirmed that the hagfish olfactory lamella exhibits a laminar structure (Figure 5C), in a manner similar to that of the teleost fish.^29^ Each lamella consists of a medial fibrous layer called the central core and the peripheral olfactory epithelium, which is further subdivided into three layers: from proximal to distal, the basal cell (BC) layer, the olfactory sensory neuron layer, and the apical cilia/microvilli layer.

Based on this observation, we then examined gene expression patterns of inshore hagfish *OR5*, *V1R1*, and *V2R33*. As a result, we found that all of them were expressed in olfactory cells in a scattered pattern (Figures 5D–5F). Negative controls using sense probes showed no such expression patterns (Figure S4). Among these genes, *V1R1*-positive cells showed a distinctive pattern, being broadly and relatively densely distributed in the olfactory sensory neuron layer (Figure 5E). In addition, some *V1R1*-positive cells were also observed in the BC layer. In contrast, the olfactory cells expressing the other two genes (*OR5* and *V2R33*) were more sparsely distributed and found in a relatively superficial part of the olfactory sensory neuron layer (Figures 5D and 5F).

## Discussion

### Hagfish olfactory repertoire

**Figure 6:**
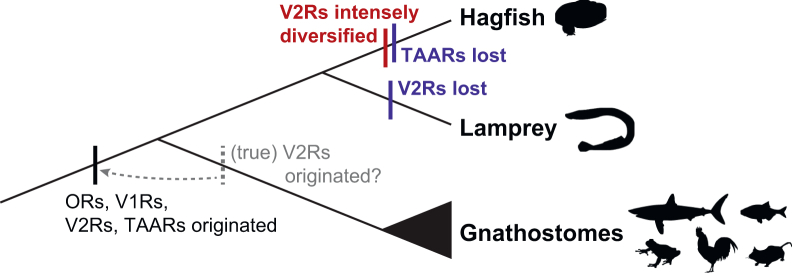
Diversification of olfactory repertoire in vertebrate evolution Silhouettes from PhyloPic (public domain).

In this study, we have investigated the olfactory receptor repertoire in hagfish. By combining genome-wide and gene expression analyses, we identified putatively functional olfactory repertoire genes as follows (see also Table 1 and Figure 6).

#### ORs

We identified 48 inshore hagfish *OR*s, all of which fall into type 1 *OR*s (Figure 1A). In addition, almost all of them (47 genes) are highly expressed in the olfactory organ (Figure 2) and at least one of them (*OR5*) in olfactory sensory neurons (Figure 5D), suggesting that these genes encode functional (i.e., “true”) *OR*s. As these inshore hagfish ORs form a monophyletic group together, these genes are considered to have duplicated through hagfish-specific diversification.

Nevertheless, no inshore hagfish *OR*s were classified as type 2 subfamily. In contrast, we showed the lamprey has both type 1 and type 2 subfamilies consistent with previous reports,^6^ suggesting that the common ancestor of all vertebrates had both types, but hagfish has lost type 2 *OR*s.

#### V1Rs

In this study, we found only two inshore hagfish *V1R*s, which show high affinity to the *ORA3/4* subfamily and *ORA5* subfamily, respectively (Figure 1B). We also demonstrated that these genes are expressed in the olfactory organ and at least one of them (*V1R1*) is expressed in olfactory sensory neurons (Figures 4A and 5E). These results suggest that hagfish have true *V1Rs*.

Still, we identified more *V1R*s (eight genes) from the latest version^13^ of the sea lamprey genome. These genes include two additional ones that were unreported in previous research.^9^ Among them, two (lamprey *V1R1* and *V1R2*) show affinity to the gnathostome *ORA1/2*, two (lamprey *V1R3* and *V1R7*) to the gnathostome *ORA3/4*, and one (lamprey *V1R6*) to *ORA6*. The remaining three (lamprey *V1R4*, *V1R5*, and *V1R8*) were placed in the basal position of the gnathostome *ORA5/6*, despite showing low support values. Combining these results with previous research,^9^ it is suggested that the common ancestor of all vertebrates had at least three subtypes of *V1R*s, *ORA1/2*, *ORA3/4,* and *ORA5/6*, and that the hagfish has lost the *ORA1/2* gene(s).

#### V2Rs

Most notably, we revealed that *V2R*s show the highest diversification among hagfish olfactory repertoire genes. We identified typical *V2R*s (135 genes) that form a monophyletic clade basal to the gnathostome *V2R*s. As the closest outgroup to these *V2R*s, we found a pair of *V2R-like* genes in the inshore hagfish and the sea lamprey. Consistent with a previous suggestion that lamprey *V2R*s do not function for olfaction,^9^ our transcriptome analysis indicates that the inshore hagfish *V2R-like* gene is not expressed in the olfactory organ at a functional level (Figure 4B).

In contrast, we confirmed that all of the true *V2R*s of the inshore hagfish are expressed in the olfactory organ (Figure 4B), particularly and at least one of them (*V2R33*) in olfactory sensory neurons (Figure 5F), suggesting that these genes do serve as olfactory repertoire genes. This finding challenges the conventional view that the olfactory function of the *V2R*s was established in the gnathostome lineage.^9^^,^^12^ Instead, we suggest that true *V2R*s had already been acquired in the common ancestor of all vertebrates; these genes underwent extensive diversification in hagfish, but they have been lost specifically in the lamprey lineage.

#### TAARs

We found no *TAAR* gene candidates in the current version of the inshore hagfish genome, consistent with previous research.^24^ In contrast, previous studies^8^^,^^10^ reported that the lamprey has dozens of *TAAR*s. Therefore, it is suggested that *TAAR*s can be traced back to the common ancestor of all vertebrates and that they have been lost specifically in the hagfish lineage.

### Importance of assembly quality for comparative genomics

To obtain a more complete gene list for comparison, we re-surveyed lamprey olfactory repertoire genes using the latest version^13^ of the sea lamprey genome. As a result, we identified a greater number of *OR* and *V1R* genes than previously reported (Table 1), suggesting that better genome assemblies provide more reliable information for comparative genomics.

As the current version of the inshore hagfish genome used in this study is not completely assembled at the telomere-to-telomere level,^30^ future high-quality assemblies may reveal additional olfactory repertoire genes that are not detected in this study. In addition, if a complete genome assembly were available, our analysis of the genomic distribution of hagfish olfactory repertoire genes would be more precise. Still, the overall trends in their diversification, loss, and genomic distribution are clearly demonstrated.

### Gene duplication of hagfish olfactory repertoire genes

Our analysis of the genomic mapping of hagfish olfactory repertoire genes revealed that phylogenetically close *OR* and *V2R* genes tend to reside close in the hagfish genome. Particularly in the scaffold FYBX03000002.1, phylogenetically close *V2R* genes were organized in clusters along the genome. However, a given genomic cluster often contains genes from different clades (Figure 2B). This distribution pattern suggests that these genes have been diversified through tandem gene duplications and chromosomal rearrangements, as has been observed in human olfactory repertoire gene evolution.^31^

It is suggested that whole-genome duplication (WGD) may contribute to the genetic diversity of the olfactory repertoire genes.^32^ Recently, the cyclostomes were proposed to have experienced lineage-specific WGD or triplication.^30^^,^^33^ Nevertheless, this event appears to have had no or a small contribution to the evolution of olfactory repertoire genes, especially *V2R*s. In fact, the scaffold FYBX03000002.1 includes most of the inshore hagfish *V2R* genes (91%), indicating that these genes have duplicated at the intrachromosomal level.

Another possible mechanism is retrotransposon-mediated duplication. However, this scenario is unlikely in the diversification of hagfish *V2R*s because retrotransposon-encoded reverse transcriptase typically generates intronless genes,^34^ and all inshore hagfish *V2R*s contain introns except for a few exceptions whose domains were only partially predicted (Table S1).

Taken together, the most plausible mechanism for the extensive diversification of hagfish *V2R*s is tandem gene duplications followed by chromosomal rearrangements.

### Expression specificity of the olfactory repertoire genes

In mammals, it is widely believed that each olfactory sensory neuron expresses only one receptor gene.^35^ This “one neuron-one receptor” hypothesis was originally proposed based on studies in mice and rats (e.g., Chess et al.^36^ and Malnic et al.^37^). Nevertheless, it has yet to be conclusively demonstrated.^38^ In fact, a previous study showed that two functional odor receptors are expressed in one neuron in the fruit fly.^39^

Particularly, whether the “one neuron-one receptor” rule also applies to basal vertebrates remains an open question. The broad expression pattern of the *V1R* gene in the inshore hagfish olfactory epithelium (Figure 5E) implies the possibility that *V1R*s are coexpressed with other olfactory repertoire genes in a single olfactory sensory neuron. To test this hypothesis in cyclostomes, further analyses—such as single-cell RNA-seq—are necessary.

### Cell morphology and distribution of the olfactory repertoire gene-expressing neurons

Certain vertebrates exhibit lineage-specific distribution patterns of olfactory sensory neurons. For example, mammals have two distinctive olfactory organs: the main olfactory organ and the vomeronasal organ. The former consists of *OR*- and *TAAR*-expressing ciliated cells, while the latter is specialized for pheromone detection and contains *V1R*- and *V2R*-expressing—and additionally in rodents,^40^ formyl peptide receptor (*FPR*)-expressing—microvillous cells.^41^ In teleost fish, there are four types of olfactory sensory neurons: *OR*- and *TAAR*-expressing ciliated cells, *V1R*- and *V2R*-expressing microvillous cells, *V1R*-expressing crypt neurons, and kappe neurons with unidentified receptors.^41^ Among them, the *OR*-expressing ciliated cells are found in the deep layer of the olfactory epithelium, while the *V1R*/*V2R*-expressing microvillous cells, along with crypt neurons, are located in the superficial layer.^23^^,^^42^

In hagfish, ciliated and microvillous cell types have been reported to be present.^28^ However, the relationship between these cell types and olfactory repertoire gene expression patterns, as well as the spatial distribution of olfactory sensory neurons, remain unresolved. Although our gene expression analysis suggests that both *OR*- and *V2R*-expressing cells are located in the relatively superficial layer (Figures 5D and 5F), further detailed analyses, including spatial RNA-seq, would provide key information for these questions.

### Lineage-specific diversification of olfaction in early vertebrates

One of the most notable findings of this study is that functional *V2R*s are present and extensively diversified in the cyclostome hagfish. While V2Rs are reported to bind to water-soluble molecules such as the peptide ligands of major histocompatibility complex class I molecules and exocrine gland peptides in mammals,^19^^,^^20^ they are also expected to detect amino acids and their derivatives, eliciting feeding behaviors in teleosts.^21^^,^^22^^,^^23^ The massive diversification of inshore hagfish *V2R*s may thus reflect an adaptation to scavenging life in low-light environments, with various functions such as the detection of food sources and mating partners. To determine the functions of hagfish V2Rs, it is necessary to examine these receptors from multiple disciplinary perspectives, including ligand-binding assays, electrophysiological recordings, and behavioral experiments using candidate odorants.

Furthermore, our phylogenetic analyses revealed that the olfactory repertoires in basal vertebrates have evolved in a lineage-specific manner (Figure 6). On the one hand, hagfish have experienced extensive diversification of *V2R*s and loss of *TAAR*s. On the other hand, lampreys appear to have lost true *V2R*s. As previous studies have relied on lampreys only, it has been mistakenly suggested that true *V2R*s originated in the common ancestor of gnathostomes. However, this study pushes the evolutionary origin of the *V2R*s back to the common ancestor of all vertebrates. This finding calls attention to the importance of the hagfish, which has often been overlooked, yet holds significant potential to illuminate critical aspects of early vertebrate evolution.

### Limitations of the study

There are also some limitations in our research. First, as mentioned in the section discussion, a more complete assembly may reveal additional olfactory repertoire genes that are not detected in this study. Second, our analysis focused on *E. burgeri* only, although species-specific diversification likely exists within the hagfish lineage. To uncover such interspecific variation, detailed comparative analyses using additional hagfish species (e.g., *Eptatretus atami* and *Myxine glutinosa*) are required. Last, the functional aspect of the hagfish olfactory repertoire and its behavioral consequences were only indirectly estimated in this study. Ultimately, our results provide a promising foundation for a more comprehensive understanding of the hagfish olfactory system, which provides key insights into the evolutionary origin of vertebrate olfaction.

## Resource availability

### Lead contact

Further information and requests for resources and materials should be directed to and will be fulfilled by the lead contact, Daichi G. Suzuki (suzuki.daichi.gp@u.tsukuba.ac.jp).

### Materials availability

This study did not generate any new physical materials.

### Data and code availability

• Data: This paper analyzes publicly available data that are listed in the key resources table.
• Code: This paper does not report original code.
• All other items: Any additional information required to analyze the data reported in this paper is available form the lead contact upon request.

## Acknowledgments

We thank Drs. Hiroshi Wada and Yoshiaki Morino for their valuable comments and Dr. Haruka Ozaki for providing computer resources. We appreciate Susumu Hatanaka (Shinsho Maru; Fujisawa, Kanagawa, Japan) and Masanori Nishizaki and Dr. Masa-aki Yoshida (Oki Marine Biological Station, Shimane University) for providing the *E. burgeri* specimens. Computations were partially performed on the NIG supercomputer at ROIS National Institute of Genetics. This work was supported by the grant-in-aid for the 10.13039/501100001691Japan Society for the Promotion of Science (JSPS; grant numbers JP20K15855, JP22K15164, JP24K09556, and JP24H01538 to D.G.S, and JP19K16178 to Y.Y.) and by the Sasakawa Scientific Research Grant from The 10.13039/501100007807Japan Science Society (grant number 2023-4098 to H. Kashima.).

## Author contributions

Conceptualization, D.G.S. and Y.Y; methodology, H. Kariyayama., Y.O., H. Kashima., T.N., and R.H.; investigation, H. Kariyayama., Y.O., H. Kashima., T.N., and R.H.; writing – original draft, H. Kariyayama. and D.G.S.; writing – review & editing, H. Kariyayama., D.G.S., and Y.Y.; funding acquisition, D.G.S., Y.Y., and H. Kashima.; resources, D.G.S. and Y.Y.; supervision, D.G.S. and Y.Y.

## Declaration of interests

The authors declare no competing interests.

## STAR★Methods

### Key resources table

**Table:** 

REAGENT or RESOURCE	SOURCE	IDENTIFIER
**Antibodies**		

Alkaline phosphatase (AP)-conjugated anti-digoxigenin Fab fragments	Roche	11093274910

**Biological samples**		

Inshore hagfish (*Eptatretus burgeri*) from Sagami Bay, Kanagawa Prefecture	This study	N/A
Inshore hagfish (*Eptatretus burgeri*) from Kamo Bay, Oki, Shimane Prefecture	Yamaguchi et al.^43^	N/A

**Chemicals, peptides, and recombinant proteins**		

T3 RNA polymerase	Roche	11031163001
T7 RNA polymerase	Roche	12352204
Tissue-Tek Optimum Cutting Temperature (O.C.T.) Compound	Sakura Finetek Japan	4583
Mayer’s hematoxylin solution	Wako	131–09665
1% Eosin Y solution	Wako	051–06515
G-NOX	Genostuff	GN04
PARAmount-D	Falma	308-500-1
0.5% blocking reagent	Roche	11277073910

**Deposited data**		

Bulk RNA-seq data of the olfactory organ of the hagfish	This study	DRR707971
Bulk RNA-seq data of the brain of the hagfish	Yamaguchi et al.^43^	DRR707969
Bulk RNA-seq data of the pituitary gland of the hagfish	Yamaguchi et al.^43^	DRR707970
Bulk RNA-seq data of the gill of the hagfish	Yamaguchi et al.^43^	DRR707972
Bulk RNA-seq data of the liver of the hagfish	Yamaguchi et al.^43^	DRR707975
Bulk RNA-seq data of the kidney of the hagfish	Yamaguchi et al.^43^	DRR707976
Bulk RNA-seq data of the intestine of the hagfish	Yamaguchi et al.^43^	DRR707977
Bulk RNA-seq data of the testis of the hagfish	Yamaguchi et al.^43^	DRR707979
Bulk RNA-seq data of the ovary of the hagfish	Yamaguchi et al.^43^	DRR707980

**Experimental models: Organisms/strains**		

Inshore hagfish (*Eptatretus burgeri*) from Sagami Bay, Kanagawa Prefecture	This study	N/A
Inshore hagfish (*Eptatretus burgeri*) from Kamo Bay, Oki, Shimane Prefecture	Yamaguchi et al.^43^	N/A

**Oligonucleotides**		

*OR5* Forward primer: CGATGTATTCTTCCT CAAAACCATGTTCCA	This study	N/A
*OR5* Reverse + T3 primer: attaaccctcactaaa gggaATGGGGTTGCTGATAGGAGG	This study	N/A
*V1R1* Forward primter: GATCATGCACCTG GCGTTGGTAAATCTGGT	This study	N/A
*V1R1* Reverse + T3 primer: attaaccctcacta aagggaCCGAGAGGCTTCACATCTCC	This study	N/A
*V2R33* Forward primter: GCATGGAAGTAT CGAACAATCACAAAGCAT	This study	N/A
V2R33 Reverse + T3 primer: attaaccctcacta aagggaAAATGAAGCACAGACCTTGC	This study	N/A

**Software and algorithms**		

Trimmomatic (Version 0.38)	Bolger et al.^44^	https://www.usadellab.org/cms/?page=trimmomatic; RRID: SCR_011848
FastQC (Version 0.11.9)	Andrews et al.^45^	https://www.bioinformatics.babraham.ac.uk/projects/fastqc/; RRID: SCR_011848
Salmon (Version 1.10.3)	Patro et al.^46^	https://github.com/COMBINE-lab/salmon; RRID: SCR_017036
SeqKit (Version 2.8.2)	Shen et al.^47^	https://bioinf.shenwei.me/seqkit/download/; RRID: SCR_018926
HMMER3 (Version 3.2.1)	Mistry et al.^48^	https://hmmer.org/; RRID: SCR_005305
BLAST+ (Version 2.16.0)	Camacho et al.^49^	https://ftp.ncbi.nlm.nih.gov/blast/executables/blast+/2.16.0/; RRID:SCR_004870
GeneWise (Version 2.4.1)	Birney et al.^50^	https://www.ebi.ac.uk/∼birney/wise2/; RRID:SCR_015054
MAFFT (Version 7.481)	Katoh and Standley^51^	https://mafft.cbrc.jp/alignment/software/linux.html; RRID:SCR_011811
TrimAL (Version 1.4.rev15)	Capella-Gutiérrez et al.^52^	https://github.com/inab/trimal; RRID:SCR_017334
RAxML (Version 8.2.12)	Stamatakis^53^	https://github.com/stamatak/standard-RAxML; RRID:SCR_006086
R (Version 4.2.2)	R Center For Statistical Computing	https://www.r-project.org/; RRID:SCR_001905
ggbio (Version 1.46.0)	Yin et al.^54^	https://lawremi.github.io/ggbio/; RRID:SCR_003313
ape (version 5.7.1)	Paradis and Schliep^55^	https://github.com/emmanuelparadis/ape; RRID: SCR_017343
Tidyverse (Version 2.0.0)	Wickham et al.^56^	https://tidyverse.tidyverse.org/; RRID:SCR_019186
ggplot2 (Version 3.5.2)	Wickham^57^	https://ggplot2.tidyverse.org/; RRID:SCR_014601

### Experimental model and study participant details

#### Animals

Inshore hagfish (*E. burgeri*) specimens, both male and female, were collected from Sagami Bay, Kanagawa Prefecture in 2021–2025 or from Kamo Bay near Oki Marine Biological Station, Shimane University (Oki, Shimane, Japan) in 2021. As for the animals from Sagami Bay, they were transported to the laboratory at the University of Tsukuba, Japan, where they were kept in 160 L tanks filled with circulating artificial seawater at 12°C ± 0.5 °C before dissection. As for the animals from Kamo Bay, they were transported to a laboratory at Matsue campus, Shimane University (Matsue, Shimane, Japan). They were maintained in a 260 L tank filled with circulating artificial seawater at 15 ± 0.5 °C and sacrificed for tissue sampling. All procedures in this study were performed in compliance with the guidelines for animal use of the Animal Care Committees at the University of Tsukuba and Shimane University (specific approval is not required for experimentation on fishes under the Japanese law, Act on Welfare and Management of Animals). During the investigation, every effort was made to minimize suffering and to reduce the number of animals used.

### Method details

#### Tissue sampling

For RNAseq of the olfactory organ, the tissue was collected from the decapitated animal following ice anesthesia, and was frozen using liquid nitrogen and kept at −80 °C until analyzed. For molecular cloning, histological analysis, and *in situ* hybridization, animals were deeply anesthetized with MS-222 (100 mg/L; Sigma, A5040) and euthanized by decapitation. Then, the decapitated heads were dissected to harvest the olfactory organs, which were served for each experiment described below.

#### RNA-seq analysis

RNA-seq of the olfactory organ was newly performed in this study, following the method for other organs described previously.^43^ Total RNAs were extracted from the frozen tissue from a single individual (total length 55 cm, weight 226.3 g, male; as for other tissues, the pituitary glands from five individuals of mixed sexes and sizes were pooled, while other tissues were collected from a single individual, as reported.^43^ Raw RNA-Seq data are available at the public repository (BioProject: PRJDB35770, DRR Run: DRR707969–DRR707972, DRR707975-DRR707977, DRR707979, and DRR707980).

The adaptor sequences and low-quality sequences were then removed using Trimmomatic version 0.38.^44^ We used FastQC version 0.11.9^45^ to evaluate the sequence data quality. The gene models of *E. burgeri* were retrieved from Ensembl Release 104 and were used as a reference for calculating expression levels. To prepare a more comprehensive reference dataset, we added sequences identified from the genome by TBLASTN searches.^49^ The expression amount was quantified as transcripts per million (TPM) using Salmon version 1.10.3.^46^ For visualizing the expression amount of olfactory repertoire genes, we used the tidyverse^56^ and ggplot2 packages^57^ in R version 4.2.2 (R Center For Statistical Computing, Vienna, Austria). TPMs were log10-transformed after adding a pseudocount of 0.001.

#### Gene search

To identify olfactory receptor (*OR*) gene candidates, we performed BLASTP searches^49^ on the hagfish and sea lamprey (*P. marinus)* gene models using *OR* sequences of gnathostomes as queries. The gnathostomes included the following species: human, mouse, zebrafish, and African clawed frog. We retrieved hagfish gene models and genome sequences from Ensembl (Release 104). As a new version of the sea lamprey genome was reported recently,^13^ we re-surveyed *OR*s of this animal. The amino acid (AA) sequences of the gnathostomes were retrieved from Niimura and Nei.^58^ We extracted inshore hagfish and sea lamprey sequences from the BLAST search result with E values < 1E-3 and an alignment length of ≥ 250 AA. To confirm the homology, we also conducted BLASTP searches against the human, zebrafish, and African clawed frog gene models (Ensembl release 104) using the hagfish and sea lamprey sequences as queries with the same thresholds. For gene extraction, we used SeqKit version 2.8.2.^47^ To narrow down the list to more plausible sequences, we performed a domain search using HMMER 3 version 3.2.1^48^ with the Pfam database version 34.0.^59^ As a result, we found 35 genes from the hagfish and 61 genes from the sea lamprey gene models.

Recently, a new version of the inshore hagfish genome was published,^30^ but gene models based on this genome data are not officially provided. To extract potential *OR* gene candidates that were missed in the gene models based on the old version, we searched for *OR* candidate genes directly from the new one. To identify potential *OR* gene candidates, TBLASTN was performed on the hagfish genome version 4.0 (GenBank: GCA_900186335.3). We used the same query sequences as the analysis using gene models. The threshold for homologous sequences was E value < 1E-10 and alignment length ≥ 250AA as described in the previous research.^58^ To confirm their homology, we also conducted BLASTP searches as described above. The coding sequences were predicted from TBLASTN hit regions with 900 bp of flanking sequences using GeneWise version 2.4.1^50^ with default parameters. We used the same query sequences for this TBLASTN search as those used for the BLASTP search described above. Next, the possibly functional sequences were extracted from the predicted sequences using HMMER 3 and the Pfam database. 29 sequences were matched to the *OR* candidate genes extracted from the gene models, and 13 sequences were newly identified from the genome sequences.

We collected candidate sequences of hagfish *V1R* and *V2R* genes in the same way as for *OR*s. In the reciprocal BLAST search of gene models, homologous genes of *V2R*, *CaSR*, taste receptor type 1 (*TAS1R*), and G protein-coupled receptor family C group 6 (*GPRC6*) were extracted as the candidate sequences for *V2R*s. The plausible sequences were extracted with at least 200 AA of a 7-transmembrane domain (PF00003, TM7_3) by domain searches for *V2R*s. As a result of BLAST searches and domain searches against the gene models, two and 38 gene models were identified as candidates of *V1R*s and *V2R*s, respectively. The 99 additional candidate sequences of *V2R*s were found from the hagfish genome in a similar way to identify *OR* sequences. To predict coding sequences by GeneWise, we used sequences found by TBLASTN against the TM7 domain. As *V2R*s contain another domain in their N-terminal region, we also included this domain and the intron sequences for prediction if a TBLASTN hit sequence is found to contain it (on the same strand and intersequences between the TBLASTN hits < 100,000 bp). In the other case, that is, even if a TBLASTN hit sequence does not include the N-terminal region, we also used any such sequence for our analysis because there is a possibility that its N-terminal domain is just not detected. In addition, we identified one and 11 more plausible sequences of *V1R* and *V2R*s, respectively, that correspond to the gene models. Thus, we used 110 predicted genes, including the 12 sequences for downstream analysis.

For lamprey *V1R* genes, we initially identified four genes from the sea lamprey (*P. marinus*) gene models. While three genes were previously reported,^9^ the other one (*P. marinus* *V1R7*) was newly identified. Because the detected genes were fewer than *V1R*s reported in the previous study, we further conducted a TBLASTN search against the sea lamprey genome. As a result, we detected seven *V1R* candidate sequences (six known *V1R* genes and *P. marinus* *V1R7*). Additionally, we searched *V1R* sequences against the arctic lamprey (*L. camtschaticum*) genome and gene models.^60^^,^^61^ We detected the seven genes, of which five correspond to the genes identified in the previous research. Although another gene corresponded to *P. marinus* *V1R7*, it was truncated. One of the seven genes (*L. camtschaticum* *V1R8*) was newly identified, and we also found the orthologous gene (*P. marinus* *V1R8*) from the sea lamprey genome. In summary, we identified eight lamprey *V1R* genes for the phylogenetic analysis.

#### Phylogenetic analysis

We retrieved AA sequences of the gnathostome and amphioxus ORs from Niimura,^6^ V1R and TAS2R sequences from the Ensembl database and previous research,^9^^,^^14^^,^^62^^,^^63^ and V2R, TAS1R, GPRC6, CaSR, glutamate receptor metabotropic (GRM), and gamma-aminobutyric acid type B receptor (GABBR) sequences from previously published papers and the ENSEMBL database.^9^^,^^12^ These sequences were aligned using MAFFT version 7.481^51^ with the default option and trimmed to poorly aligned regions using TrimAL version 1.4. rev15^52^ with the “gappyout” option. Phylogenetic analysis was conducted using RAxML version 8.2.12^53^ with the options of “-f a -x 12345 -p 12345 -# 500 -m PROTGAMMAAUTO --auto-prot=aic -T 16.”

#### Genomic mapping and distribution analysis

To classify subgroups of *OR* genes and *V2R* genes, we focused on clades containing at least five genes with their bootstrap value ≥ 85%. We aimed to obtain three or more clades for ORs and V2Rs, respectively, trying to define as larger clades as possible at the same time. Consequently, we obtained three clades for *OR*s and nine clades for *V2R*s (Figures S1 and S2). The *V2R* genes that were not classified into any of these clades were categorized into “Else” (Figure S2). We then visualized the genomic distribution of the *OR* genes using ggbio version 1.46.0^54^ within R, based on this classification.

For scatter plotting, we calculated the phylogenetic and genomic distances for all interclade and intraclade pairs of the mOR genes and of the *V2R* genes, respectively, based on the clade classification. We included the pairs of “Else” *V2R* genes into the interclade pairs. The phylogenetic distances were obtained using the “cophenetic.phylo” function in the ape version 5.7.1.^55^ Accordingly, we visualized the relationship between the genomic and phylogenetic distances using the tidyverse version 2.0.0^56^ and ggplot2 version 3.5.2.^57^

To identify the homologous genes of phospholipase C eta (*Plch*) and membrane metallo-endopeptidasemme (*Mme*)/neprilysin (*Nep*) from the inshore hagfish, we conducted BLASTP search^49^ against the gene models. For the BLASTP search of the Mme sequences, we used the human MME (NP_001341571), the mouse Mme (NP_001344264), and the tropical clawed frog Mme (XP_031758820) as the query. For the search of Plch sequences, the human PLCH1 (NP_001124432), the mouse Plch1 (NP_899014), the tropical clawed frog Plch1 (ENSXETP00000067389), and zebrafish Plch1 (ENSDARP00000119930) were used. We confirmed the homology by reciprocal BLAST search as described in olfactory repertoire gene search.

To compare microsynteny around the *Mme* gene and the *Plch* gene of the inshore hagfish, we collected flanking protein-coding genes from the genome annotations of Ensembl. We used eight genes from each of the scaffolds (FYBX02010589.1, FYBX02009490.1, and FYBX02009650.1) , and then converted these scaffolds to the new version of the inshore hagfish genome^30^ by BLASTN search with the threshold of E values < 1E-100 and identity > 99%. To identify the homologs of the flanking genes, we ran BLASTP search with E values < 1E-10 against the mouse, the tropical clawed frog, the Japanese pufferfish (*Takifugu rubripes*), and the sea lamprey gene models. We also extracted flanking genes of the *Plch* and the *Mme* genes from the genome annotations of Ensembl. At last, we visualized the microsynteny of the genes by the tidyverse package^56^ and the ggplot2 package.^57^

#### Molecular cloning and probe synthesis

Total RNAs were extracted from the harvested olfactory organs of adult *E. burgeri* using TRIzol Reagent (Invitrogen, 15596026). These RNAs were reverse transcribed into cDNAs using PrimeScript II 1st strand cDNA Synthesis Kit (Takara, 6210A) and were used as templates in the polymerase chain reaction (PCR). Inshore hagfish genes of interest were amplified by PCR using the primers listed in key resources table. We used reverse primers including the T3 promoter sequence (20 bp) to synthesize DIG-labeled RNA probes for *in situ* hybridization. As a negative control, we instead used forward primers including the T7 promoter sequence (20 bp) to synthesize DIG-labeled RNA probes for *in situ* hybridization (sequences not shown). DIG-labeled RNA probes were transcribed from PCR products using T3 and T7 RNA polymerase (Roche, 11031163001 and 12352204, respectively).

#### Hematoxylin-eosin (HE) staining

To perform histological analysis and *in situ* hybridization, the harvested olfactory organs were fixed with 4% PFA/PBS for 1 hour at room temperature (RT) or overnight at 4 C°. The fixed specimens were then rinsed with PBS, dehydrated in a graded methanol series (25%, 50%, 75%, and 100%), and stored at −20 °C.

For cryosectioning, the stored samples were rehydrated in a graded methanol series (75%, 50%, and 25% in PBS) and PBS. They were replaced with 10% and then 30% sucrose. Subsequently, the specimens were embedded in Tissue-Tek Optimum Cutting Temperature (O.C.T.) Compound (Sakura Finetek Japan, 4583). Frozen sections in 20 μm were prepared using a cryostat (Leica, CM1860) and mounted on MAS-coated slide glasses (Matsunami Glass Ind., SMAS-01).

HE staining was performed according to the conventional method after sections were washed with PBS to remove the O.C.T. compound. Namely, the sections were incubated in Mayer’s hematoxylin solution (Wako 131-09665) for 15 min and then rinsed with running tap water for 30 min. Afterward, the sections were incubated in 1% Eosin Y solution (Wako 051-06515). The stained sections were dehydrated in a graded methanol series (25%, 50%, 75%, 95%, and 100%) and xylene-equivalent solvent G-NOX (Genostuff, GN04), and mounted using xylene-free mounting medium PARAmount-D (Falma, 308-500-1).

#### Section *in situ* hybridization

For *in situ* hybridization, the sections were first washed with PBS to remove the O.C.T. compound and postfixed for 20 min with 4% PFA/PBS and rinsed with PBS. Afterward, they were prehybridized in hybridization buffer (50% Formamide, 5× SSC, 5× Denhardt’s solution, 50 μg/ml yeast tRNA, and 50 μg/ml heparin in deionized-distilled H2O) and then incubated in the hybridization buffer with 0.1 mg/ml probe overnight at 60 °C. Subsequently, the specimens were washed with the following solutions: 50% Formamide and 2× SSC (for 30 min at 60 °C, twice), 2× SSC (for 30 min at 60 °C, twice), 0.2× SSC (for 30 min at 60 °C, twice), and PBS (5 min at RT). For signal detection, the specimens were blocked with 0.5% blocking reagent (Roche, 11277073910) in PBS for 1 hour at RT and then incubated with 0.5 μl/ml alkaline phosphatase (AP)-conjugated anti-digoxigenin Fab fragments (diluted in 0.5% blocking reagent/PBS; Roche, 11093274910) overnight at 4 °C. After washing with tris-buffered saline (TBS) four times for 30 min each at RT, alkaline phosphatase signals were detected with 20 μl/ml NBT/BCIP in color development buffer (100 mM Tris HCl pH 9.5, 100 mM NaCl, and 50 mM MgCl2). The stained specimens were postfixed in 4% PFA/PBS, dehydrated in a graded methanol series (25%, 50%, 75%, 95%, and 100%) and G-NOX, and mounted using PARAmount-D. The prepared slides were examined under a microscope (Nikon, ECLIPSE Ni) and photographed using a microscope digital camera (Nikon, DS-Ri1).

### Quantification and statistical analysis

Quantification and statistical analyses used in this study are described in the relevant sections of the STAR Methods and figure legends.

## Supplemental information

Document S1. Figures S1–S4

Table S1. Details of the identified inshore hagfish genes

Table S2. Details of the identified sea lamprey genes

Table S3. Gene list used for the phylogenetic analysis

Table S4. Details of the flanking genes to the *Mme* and *Plch1* genes

Data S1. Detailed phylogenetic tree of ORs

Data S2. Detailed phylogenetic tree of V1Rs

Data S3. Detailed phylogenetic tree of V2Rs

Data S4. Phylogenetic tree of known TAARs with their close GPCR families, including the 5-hydroxytryptamine receptors 4 (5-HT4 or HTR4), adrenoceptors beta 1, and dopamine receptors (A) Overview, (B) Detailed tree for TAARs and HTR4s.
