# Supplementary material for: Hagfish olfactory repertoire illuminates lineage-specific diversification of olfaction in basal vertebrates
Source: iScience. 2025 Nov 19;28(12):114118. doi: 10.1016/j.isci.2025.114118 (PMC12741414; doi:10.1016/j.isci.2025.114118)
Supplement: Document S1. Figures S1–S4 [file mmc1.pdf]

iScience, Volume *28*

Supplemental information

Hagfish olfactory repertoire illuminates lineage-specific diversification of olfaction in basal vertebrates

Hirofumi Kariyayama, Yusuke Ooi, Hiromu Kashima, Taiki Nakanowatari, Riho Harada, Yoko Yamaguchi, and Daichi G. Suzuki

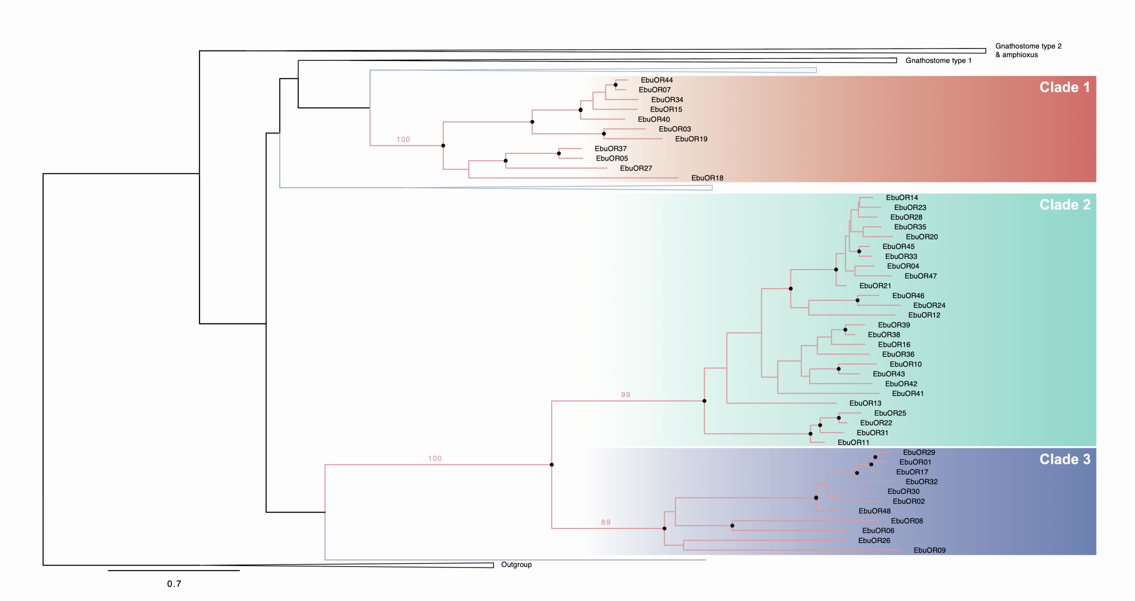

Figure S1. Classification for OR genes into Clade 1–3.

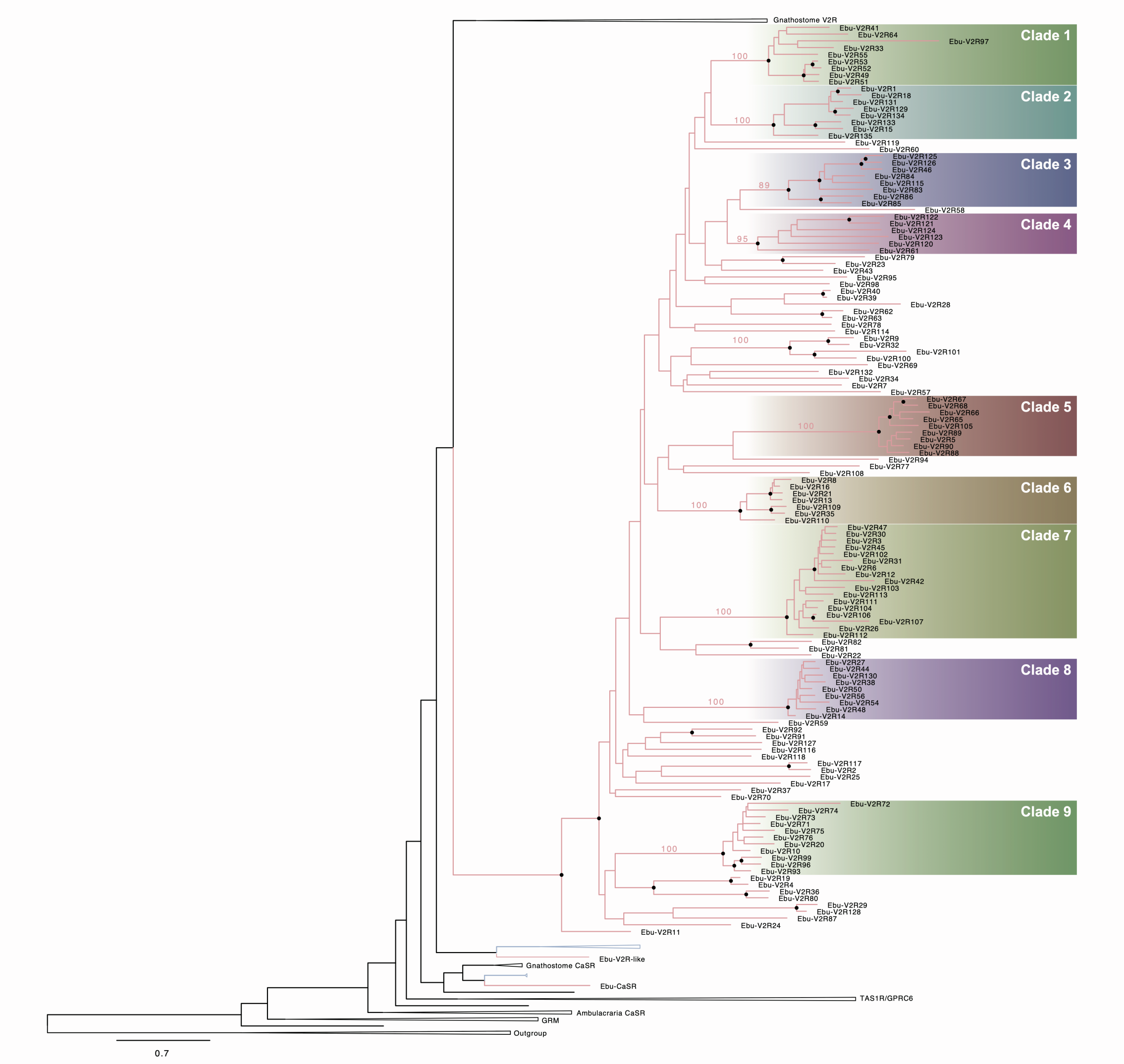

Figure S2. Classification for V2R genes into Clade 1–9.

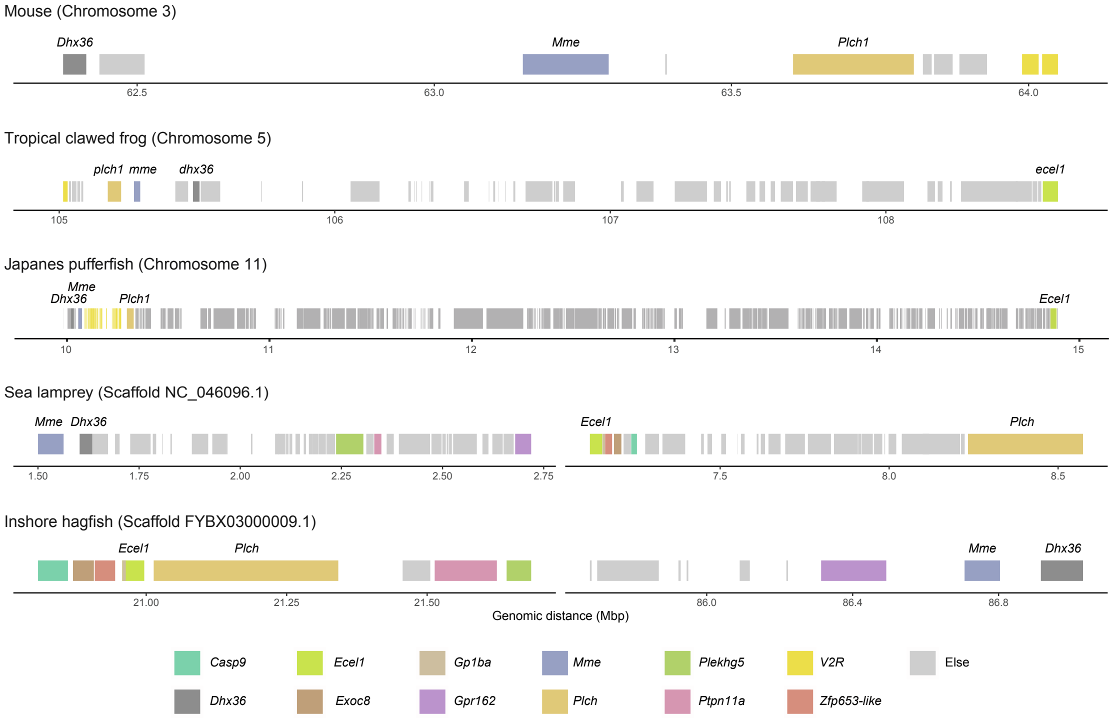

Figure S3. Microsynteny of phospholipase C eta (*Plch*) and membrane metallo-endopeptidasemme (*Mme*)/neprilysin (*Nep*).

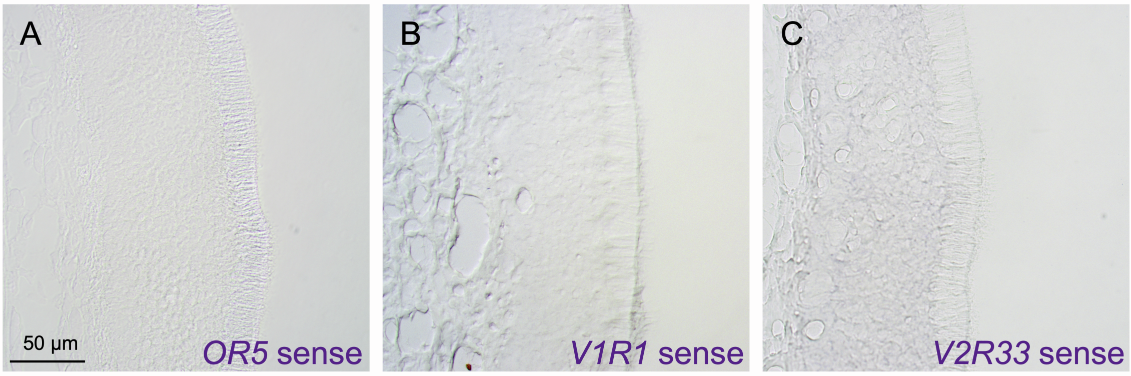

Figure S4. Negative controls for *in situ* hybridization.

Sense probes of *OR5* (A), *V1R1* (B), and *V2R33* (C). The scale bar for (A–C) is shown in (A).
