# Supplementary figures and images for: Hagfish olfactory repertoire illuminates lineage-specific diversification of olfaction in basal vertebrates
Source: iScience. 2025 Nov 19;28(12):114118. doi: 10.1016/j.isci.2025.114118 (PMC12741414; doi:10.1016/j.isci.2025.114118)

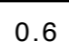

Supplement: Table S2. Details of the identified sea lamprey genes [file mmc3.pdf]

A

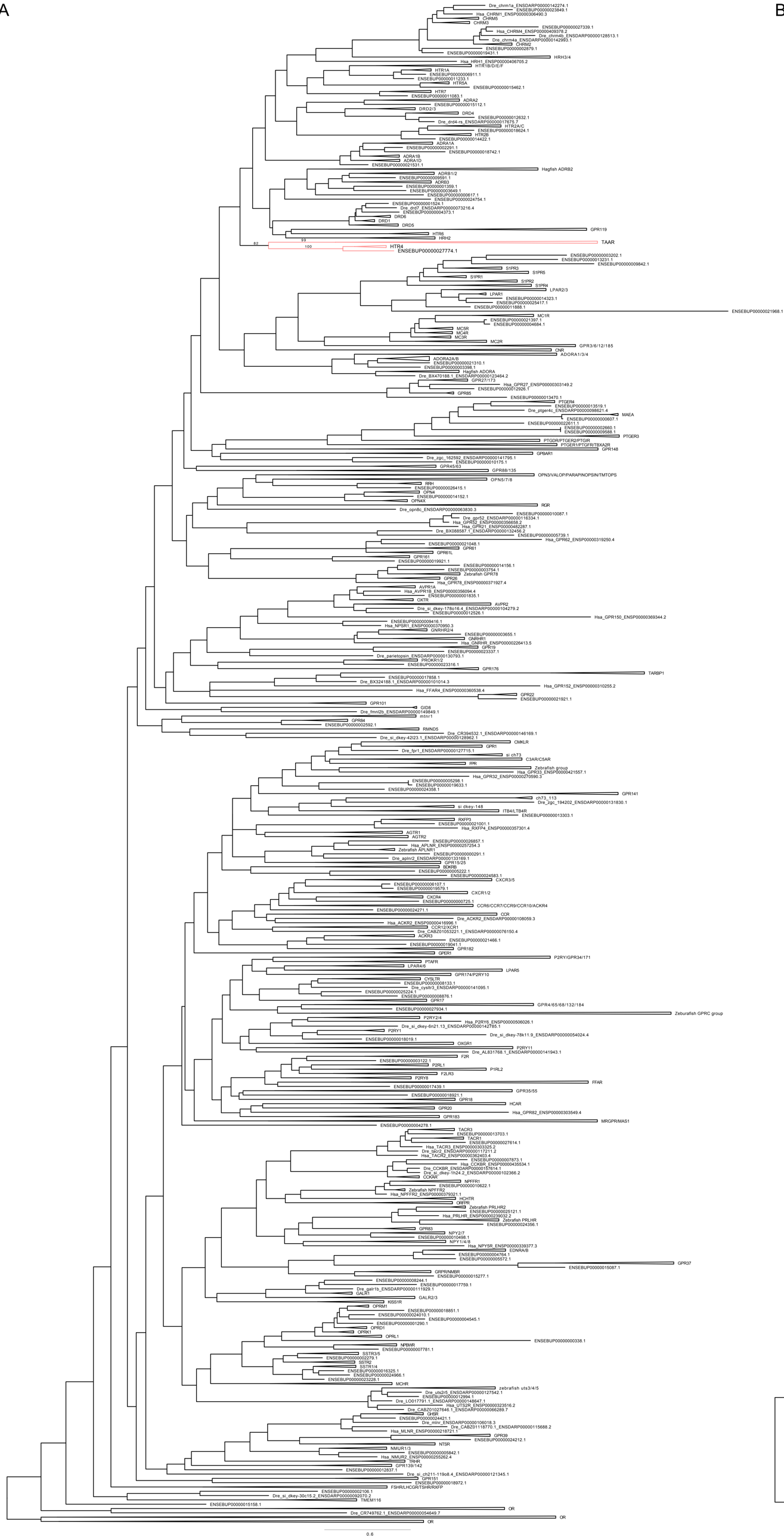

B

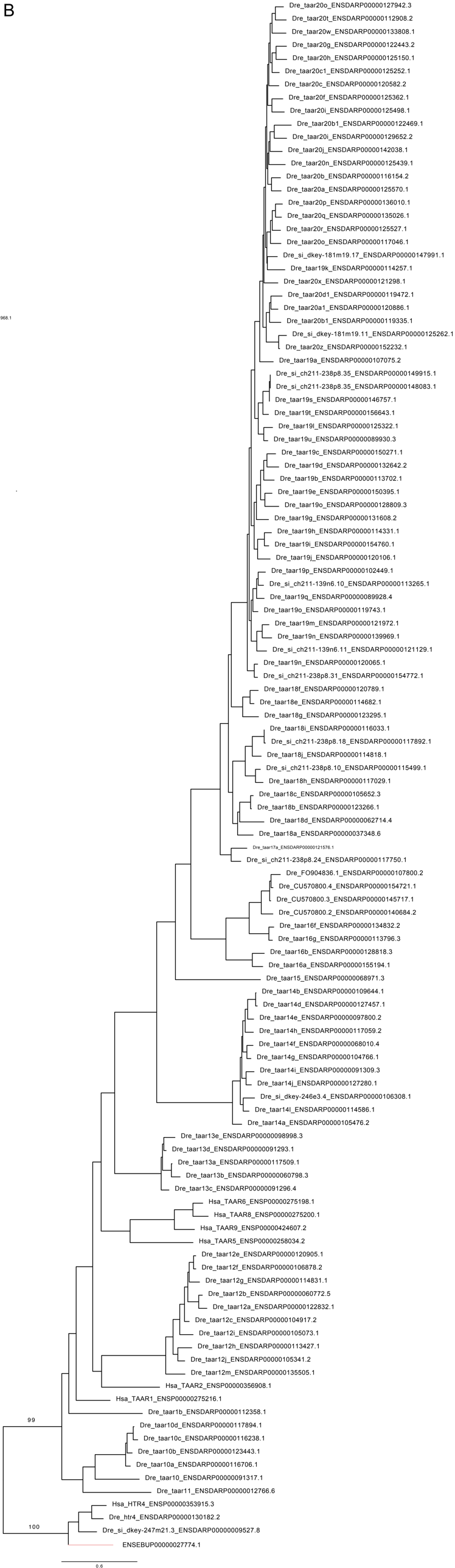

Supplement: Table S4. Details of the flanking genes to the Mme and Plch1 genes [file mmc5.pdf]
