# Supplementary material for: Hagfish olfactory repertoire illuminates lineage-specific diversification of olfaction in basal vertebrates
Source: iScience. 2025 Nov 19;28(12):114118. doi: 10.1016/j.isci.2025.114118 (PMC12741414; doi:10.1016/j.isci.2025.114118)
Supplement: Table S1. Details of the identified inshore hagfish genes [file mmc2.pdf]

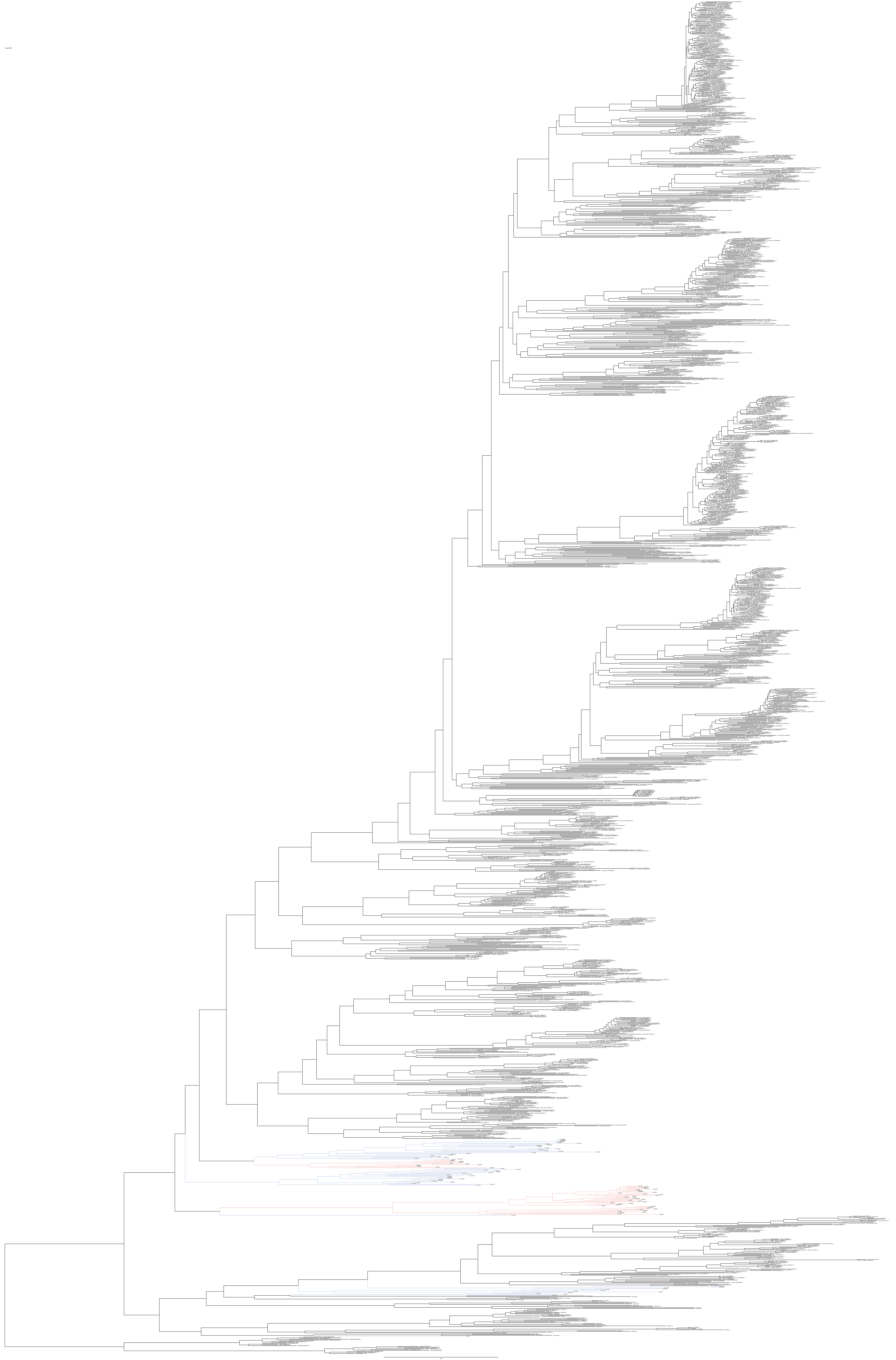
Pma-OR37
0.8
